# Index of Microcirculatory Resistance Measured during Intracoronary Adenosine-Induced Hyperemia

**DOI:** 10.1155/2020/4829647

**Published:** 2020-05-12

**Authors:** Irene Santos-Pardo, Patrik Alström, Nils Witt

**Affiliations:** Department of Clinical Science and Education, Karolinska Institute, Unit of Cardiology, Södersjukhuset, Stockholm 11883, Sweden

## Abstract

**Background:**

The index of microcirculatory resistance is an invasive measure of coronary microvascular function that has to be calculated during maximal hyperemia, classically achieved with intravenous adenosine (IV). The aim of this study was to evaluate the use of intracoronary (IC) adenosine for the calculation of IMR.

**Methods and Results:**

31 patients with stable coronary artery disease were included in the study. Coronary pressure and thermodilution measurements were obtained at rest and during maximal hyperemia using a pressure-temperature sensor-tipped coronary guidewire. Duplicate measurements were performed using first IC and then IV adenosine. Dispersion of transit times was comparable for IC and IV adenosine. IMR values based on IC vs IV adenosine showed a high level of agreement and an intraclass correlation coefficient of 0.90. Applying an upper normal limit of 25, misclassification of IMR using IC adenosine was seen in just one patient in whom IC adenosine resulted in a lower value. A simplified procedure based on a single bolus dose of saline did not change the level of agreement or the rate of misclassification.

**Conclusions:**

We found an excellent agreement between IMR values measured during hyperemia induced by IC as compared to IV adenosine. The use of IC adenosine may facilitate invasive assessment of microvascular function and is potentially time- and cost-saving with less patient discomfort as compared to IV infusion. The trail is registered with NCT03369184.

## 1. Introduction

Coronary microvascular dysfunction may be a cause of myocardial ischemia even in the absence of obstructive coronary artery disease [1]. Although these patients often present with normal noninvasive tests, metabolic evidence of ischemia has been previously shown [1–3]. A new thermodilution-based index to specifically interrogate the coronary microcirculation was postulated in 2003 [4]; the Index of Microcirculatory Resistance [5] was defined as the ratio between the distal intracoronary pressure (*P*_*d*_) and the inverse of the hyperemic mean transit time (*T*_*mn*_). Because IMR incorporates only hyperemic parameters, it eliminates the variability of hemodynamics and resting vascular tone and estimates the minimum achievable microvascular resistance [6]. Importantly, IMR is based on indirect measurements of coronary flow using *T*_*mn*,_ in contrast to Doppler-derived methods such as coronary flow reserve (CFR), based on direct measurements of coronary flow velocity. IMR has been used as a marker of microvascular dysfunction in multiple clinical scenarios such as periprocedural myocardial infarction, [7] ST-elevation infarction [8], and cardiac transplantation [9]. Previous work analyzing the microcirculation of healthy individuals suggested 25 as an upper limit of normal [10–12]. This was later confirmed in a large international registry study where IMR was calculated in 1452 coronary arteries of 1096 patients [13].

Both intracoronary (IC) and intravenous (IV) adenosine administration were shown to be capable of inducing maximal hyperemia and are therefore used for calculation of fractional flow reserve (FFR) [14] and CFR [15, 16]. Patients receiving IV adenosine commonly experience uncomfortable side effects, chest discomfort and dyspnea being the most frequent complaints [17]. Lately, IC adenosine has been more widely used in catheterization laboratories [15] due to the simple administration and the need of a lower dose of the drug, translating into a time- and cost-saving praxis. Furthermore, patient tolerance to IC adenosine is high and although not systematically studied, side effects are generally considered mild when compared to IV administration, allowing repeated measurements with minimum patient discomfort.

To our knowledge, IC adenosine has never been systematically evaluated as hyperemic stimulus for thermodilution measurements. We hypothesize that IC administration of adenosine may be used for recordings of hyperemic mean transit time, as a basis for calculation of IMR.

## 2. Materials and Methods

### 2.1. Study Population

We studied a subgroup of 31 patients included in the EPOXY-IMR study (NCT03369184), a randomized controlled trial, which aims to study the potential effect of supplemental oxygen on coronary microvascular resistance. All patients included in the study had clinical suspicion of stable coronary artery disease and at least one coronary artery with diameter stenosis 40–80% representing an indication for functional assessment. Exclusion criteria included the presence of an acute coronary syndrome, prior myocardial infarction in the past 7 days, prior heart transplantation, presence of ventricular hypertrophy as assessed by echocardiography (defined as anterior septum ≥ 13 mm in the long-axis parasternal view), severe asthma as a contraindication for the use of IV adenosine infusion, the presence of an advanced atrioventricular block in patients without prior implantation of a pacemaker, and the presence of hypoxia (defined as oxygen saturation lower than 90%). The study was approved by the regional ethics committee, and written informed consent was obtained in all patients prior to the angiography.

### 2.2. Catheterization Protocol

Cardiac catheterization was performed following the standard procedure in our catheterization laboratory; radial access was preferred if no contraindications existed, and all patients were on chronic treatment with acetylsalicylic acid or were given a loading dose of 300 mg the day before the procedure. Routine angiography cines were acquired for complete anatomic evaluation. Stenosis severity was visually assessed and based on the opinion of 2 experienced operators. Six French guide catheters were used for all measurements, and anticoagulation treatment (0.75 mg/kg enoxaparine or 50–100 E/kg unfractioned heparin) was administrated before introducing the pressure wire in the coronary artery.

### 2.3. Thermodilution Measurements and Calculation of IMR

Patients were evaluated with a pressure-temperature sensor-tipped coronary guidewire (Pressure Wire X (Abbott/St.Jude Medical, St Paul, MN, USA) with wireless technology. Pressure and thermodilution recordings were digitally stored and analyzed using the CoroFlow™ software (Coroventis Research, Uppsala, Sweden).

The aortic pressure transducer was zeroed to air, and after routine preparation and calibration, the pressure wire was introduced in the guide catheter and positioned with the wire sensor at the guide tip for electronic equalization. The pressure wire was then advanced to the distal part of the coronary artery (approximately two-thirds of the vessel), and 100 to 200 *μ*g of intracoronary nitroglycerin was administered.

**Figure 1:**
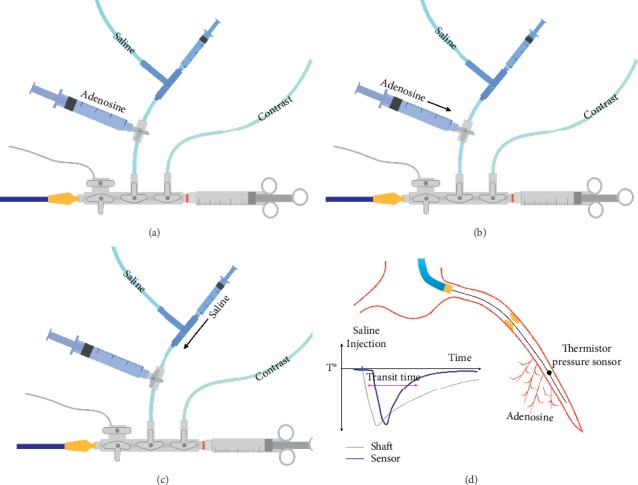
Practical setup for the measurement of IMR with IC adenosine. (a) In order to facilitate administration of IC adenosine and repeated bolus injections of saline, a closed system with a three-way stopcock was used to connect the manifold to a reservoir bag with heparinized saline and a 3 cc syringe, leaving the third connection for administration of IC adenosine. (b) Hyperemia was induced by IC administration of adenosine followed by a quick switch of the three-way stopcock in the direction of the saline reservoir and syringe. (c) Thermodilution curves were obtained by briskly injecting 3 ml of room temperature saline through the guiding catheter. Hyperemia was monitored by visual assessment of the Pd/Pa ratio throughout the procedure. Administration of IC adenosine was repeated if considered necessary to maintain maximal hyperemia. (d) The panel shows the position of the wire's thermistor/pressure sensor in the distal two-thirds of the coronary vessel. The temperature drop following a saline bolus injection is registered by the guide wire shaft as well as the tip sensor, triggering measurement of mean transit time. Abbreviations: IMR, index of microcirculatory resistance; IC, intracoronary adenosine.

During baseline conditions, simultaneous measurements of the mean aortic pressure (*P*_*a*_) and the mean distal coronary pressure (*P*_*d*_) from the pressure wire's sensor were recorded. Thermodilution curves were obtained by briskly injecting 3 ml of saline at room temperature through the guiding catheter and this was repeated 3 times, allowing for calculation of the mean transit time (*T*_*mn*_) as previously described [4]. Hyperemia was then induced by intracoronary administration of adenosine (100 *μ*g in the RCA and 200 *μ*g in the left coronary artery). After confirmation of stable hyperemia, thermodilution curves were obtained following the same procedure as described for baseline conditions. In order to rapidly perform the three saline bolus injections during IC adenosine hyperemia, a three-way stopcock system was used connecting the manifold to a reservoir bag with heparinized saline and a 3 cc syringe, leaving the third connection for administration of adenosine (Figure 1). This setup allows an immediate switch from adenosine injection to saline bolus injections. Special attention was paid to the presence of stable hyperemia throughout the complete series of injections. The presence of maximal hyperemia was assessed visually when hyperemic *P*_*d*_/*P*_*a*_ ratio achieved the typical plateau phase, shortly after the end of the flushing artifact. Generally, saline bolus injections resulted in a very short interruption of the hyperemic plateau phase in the *P*_*d*_/*P*_*a*_ recording. If the operator assessed that stable maximal hyperemia was not present at the moment of the second or third saline injections, an extra dose of IC adenosine was administered prior to continuing the procedure. Whenever this was required, adenosine was injected less briskly than the saline bolus, avoiding the triggering of a transit time measurement. After returning to baseline conditions, pressure-dilution and thermodilution measurements were repeated in the same manner as above. Hyperemia was then induced again using an IV adenosine infusion of 140 *μ*g/kg through a large cubital or femoral vein. When stable maximal hyperemia was considered present, final pressure-dilution and thermodilution measurements were performed following the same procedure as above. Calculation of IMR in vessels with functionally nonsignificant lesions (FFR > 0.80) was performed as previously described using the formula: *P*_*d*_ × *T*_*mn*_ [4]. In vessels with functionally significant lesions (FFR ≤ 0.80), IMR was calculated by adding wedge pressure measured during balloon inflation (*P*_*w*_) into a correction formula: *P*_*a*_ × *T*_*mn*_ × [*P*_*d*_ − *P*_*w*_/*P*_*a*_ − *P*_*w*_] [18]. Whenever *P*_*w*_ was not available, the formula validated by Yong et al. [19] was used instead: *P*_*a*_ × *T*_*mn*_ × [1.35 × [*P*_*d*_/*P*_*a*_] − 0, 32].

In order to assess repeatability of measurements, basal and hyperemic recordings of pressure and transit time with IC and IV adenosine were performed a second time in a subset of 22 patients.

Stenoses presenting with FFR ≤ 0.80 were treated with PCI, coronary artery by-pass surgery, or optimal medical therapy as considered appropriate by the performing physician. Functionally, nonsignificant lesions were deferred from PCI.

### 2.4. Statistical Analysis

According to its distribution, continuous data are summarized as mean and standard deviation (SD) or as median and interquartile range (25^th^–75^th^). Categorical data are presented as absolute count and percentage. In order to assess the level of agreement between IMR calculated using IC and IV adenosine, respectively, a Bland–Altman plot was created and the intraclass correlation coefficient was calculated. A Bland–Altman plot and intraclass correlation coefficient were also used to assess the agreement between the transit time obtained with the first saline injection in IC adenosine and IV *T*_*mn*_ and the agreement for the IMR value calculated with the first transit time obtained with IC adenosine compared to IMR calculated with *T*_*mn*_ during IV adenosine. A Mc Nemar's test was used to compare the differences between groups in classifying IMR as pathologic (>25). Standard deviation (SD) for the three transit time measurements during hyperemia was calculated for each patient and was normally distributed both for IC and IV adenosine. Coefficient of variation was calculated to standardize the spreading of transit times for IC and IV adenosine. A dependent Student's *T*-test was used to compare the spreading of transit times between groups. A Wilcoxon signed rank test was used to compare pairs of transit times (first and second, second and third, first and third) as these data did not distribute normally.

A two-sided *p* value of less than 0.05 was considered statistically significant. All analyses were performed using IBM SPSS Statistics software, version 23.0. Armonk, NY: IBM Corp.

## 3. Results

Baseline clinical characteristics are displayed in Table 1. In 48% of the cases, stenoses were functionally significant (FFR ≤ 0.80). However, no patient had an FFR lower than 0.50 in the studied vessel. Of those with FFR ≤ 0.80, PCI was performed *ad hoc* in 12 patients. The remaining three cases were staged due to complex stenoses and the revascularization alternatives were discussed in the Heart Team. In one patient, a coronary by-pass operation was recommended and performed. In the two remaining patients, PCI was performed some weeks later in a planed procedure. The wedge pressure was directly measured by balloon occlusion in 9 of the 15 patients who underwent PCI.

**Figure 2:**
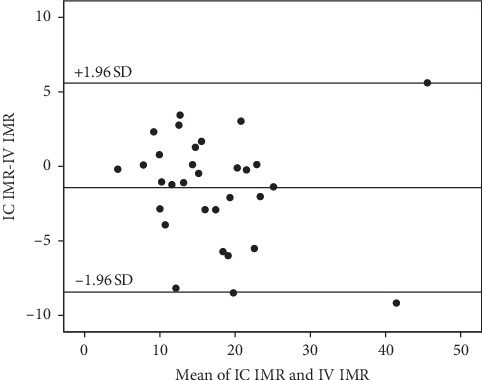
Bland Altman diagram for the primary analysis. Legend: Agreement between IMR obtained with IC compared to IV adenosine. Abbreviations: IMR, index of microcirculatory resistance; IC, intracoronary adenosine; IV, intravenous adenosine; SD, standard deviation.

In 7 of the 31 cases, an extra bolus of IC adenosine was considered needed in order to ensure that the measurements were performed during maximal hyperemia. The median procedure time was measured between start of IV infusion or first bolus injection of adenosine, and the confirmation of a successful third saline bolus injections was 148 s (range 70–308 s) and 34 s (range 28–73 s), respectively. The Bland–Altman diagram for IMR obtained with IC and IV adenosine showed a high level of agreement (Figure 2) with no signs of systematic bias. The intraclass correlation coefficient was 0.90 with a 95% confidence interval (CI) of 0.77–0.95.

**Figure 3:**
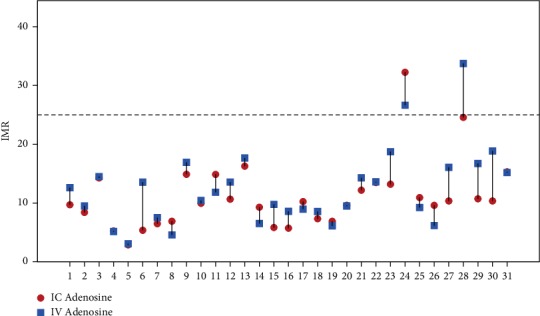
Scatter Plot representing IMR values measured with IC and IV adenosine and their relationship with the cutoff value for each subject. Legend: IMR values higher than 25 are considered pathologic. Abbreviations: IC, intracoronary adenosine; IV, intravenous adenosine; IMR, index of microcirculatory resistance.

Except for one case (where *T*_*mn*_ and IMR were lower with IC adenosine), there were no differences in classification of IMR as pathological (>25) when calculated with IC or IV adenosine (Figure 3).

*T*_*mn*_ and SD of the three transit times with IC and IV adenosine for each subject are depicted in Table 2.

No statistically significant differences in the spreading were found when comparing the transit times obtained with IC and IV adenosine (coefficient of variation for transit times 0.50 for IC adenosine and 0.47 for IV adenosine, *p*=0.210). When comparing the median value of each transit time with the others (first, second and third), no statistically significant differences were found in any pair neither for IC nor IV.

**Figure 4:**
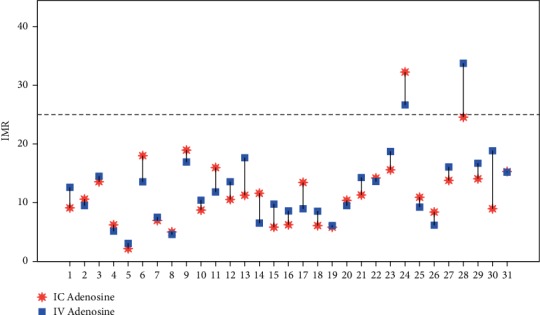
Scatter Plot representing IMR calculated with the transit time value obtained from the first injection of saline during IC adenosine-induced hyperemia as compared to the IMR value obtained with T_*mn*_ during IV adenosine. Legend: IMR higher than 25 is considered pathologic. Abbreviations: IMR, Index of Microcirculatory Resistance; T_*mn*_, mean transit time; IC, intracoronary adenosine; IV, intravenous adenosine.

When comparing the first transit time obtained with IC adenosine with the *T*_*mn*_ (average of 3) obtained with IV adenosine, the Bland–Altman diagram showed a good agreement (Supplementary material Figure S.1) that was confirmed with an intraclass correlation coefficient of 0.88 95% CI 0.75–0.94. The IMR value calculated with the first transit time obtained with IC adenosine compared to IMR calculated with *T*_*mn*_ (average of 3) during IV adenosine infusion showed an excellent agreement as shown in the Bland–Altman diagram (Supplementary Material Figure S.2), as well as by the intraclass correlation coefficient of 0.90 with 95% CI 0.80–0.95. When classifying IMR as normal or pathological (IMR > 25) with IC compared to IV adenosine-induced hyperemia, one individual was misclassified, having shorter mean transit time and lower IMR with IC adenosine (Figure 4). The same subject was misclassified when IMR was calculated with the first transit time after IC adenosine as compared with IV infusion.

In the repeatability analysis, a good agreement was shown for repeated measurements of *T*_*mn*_ during hyperemia induced by IC adenosine with an ICC of 0.97 (95% CI 0.93–0.99) as well as for IMR with an ICC of 0.87 (95% CI 0.69–0.95). The ICC for repeatability of *T*_*mn*_ with IV adenosine was 0.68 (95% CI 0.18–0.88) and 0.87 (95% CI 0.64–0.95) for IMR. There were no statistically significant differences in *P*_*d*_ during hyperemia in repeated measures with IV adenosine (*p*=0.1), a factor which could potentially have explained higher variability of IMR in the IV adenosine repeated measures subset.

**Figure 5:**
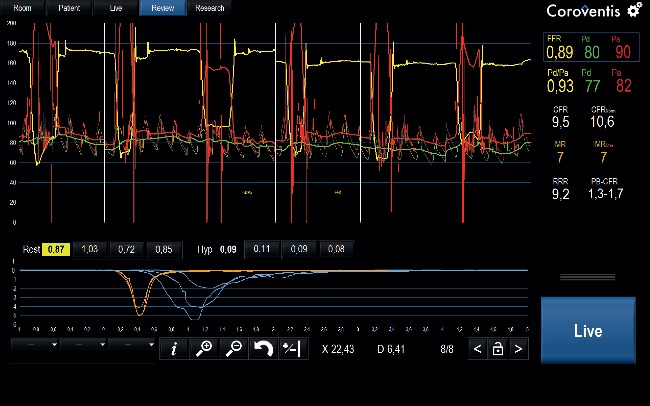
Coronary pressure and thermodilution measurements in CoroFlow™ Software (Coroventis Research, Uppsala, Sweden). Legend: Thermodilution measurements performed in the right coronary artery using intracoronary adenosine to achieve hyperemia.

Coronary pressure and flow measurements including *T*_*mn*_, CFR and IMR for each subject are displayed in Table S.1 in the Supporting Information. The three individual transit times for each patient at baseline and during hyperemia are compiled in Table S.2 in the Supporting Information.  Figure 5 exemplifies the coronary pressure and flow measurements with IC adenosine using the Coroflow™ system.

## 4. Discussion

In the present analysis, we evaluate the use of IC adenosine as hyperemic stimulus during intracoronary thermodilution measurements for the calculation of IMR. Our results show a high level of agreement between measurements using IC as compared to IV adenosine, both for transit times and for the subsequently calculated IMR values. Duplicate measurements with IC adenosine show an excellent repeatability. Furthermore, agreement was excellent in the classification of IMR as pathologic or normal when calculated with IC as compared to IV adenosine. Restricting the measurements to a single bolus injection of saline during hyperemia induced by IC adenosine did not seem to affect the level of agreement compared to standard measurements using *T*_*mn*_ during IV adenosine infusion.

The use of IC adenosine for measurement of FFR has gained popularity among interventional cardiologists and has become clinical routine in many centres [20]. Potential advantages compared to IV adenosine include shorter preparation and procedure times, lower total dose of adenosine, and potentially less patient discomfort [15]. Thermodilution measurements, however, differ from measurements of FFR in that they require a longer plateau phase of stable maximal hyperemia, also representing the major challenge of our analysis.

The duration of steady-state hyperemia after IC adenosine injection has been previously described for the standard adenosine doses used in our study, being 12 ± 13 s for a 100 *μ*g dose in the RCA and 21 ± 6 s for 200 *μ*g dose in the left coronary artery [15], leaving a reasonable time interval for repetition of 3 saline bolus injections. Given the very short half-life and good tolerance of IC adenosine, even repeated administration can be considered if needed. In the present study, a second injection of IC adenosine was considered necessary in 22% of the cases. Furthermore, as duration of hyperemia seems to be dose-dependent [15], it could be speculated that higher doses than the standard recommended would allow multiple transit time measurements after a single bolus injection of IC adenosine in most patients. When first described, IMR was calculated during hyperemia induced by IC papaverine [4]. Although IV adenosine has to be considered the current gold standard for maintaining maximal hyperemia, some potential pitfalls need to be highlighted. First, stable concentration and effect of any IV administered drug are dependent on adequate venous access. A suboptimal route of administration may result in a lower level of clinical effect, not least when half-life of the drug is very short. Therefore, a large cubital vein or femoral access is highly recommended for IV administration of adenosine. Second, the response to IV adenosine may differ on an individual basis where stability and duration of and time to maximal hyperemia may vary considerably. In a previously published systematic analysis, three different patterns of hyperemic response to IV adenosine were described. In approximately 40% of the cases, the authors found a noncontinuous hyperemic response with fluctuations in the level of hyperemia producing a multiphasic pattern where short periods of nonmaximal hyperemia alternate with a maximal hyperemia steady state. In one third of the cases, the hyperemia pattern differed between repeated measurements within a subject. Furthermore, no baseline clinical or physiological characteristics were found to predict a specific pattern of hyperemic response [16]. It is not known whether this variability has any important effect on the calculation of IMR, but attention should be paid to the relation between hyperemic response and the timing of saline bolus injections. Interestingly, we found a lower repeatability for IV as compared to IC measurements suggesting a wider variation of hyperemia between repeated measurements during IV adenosine infusion. Other factors such as hemodynamic parameters are unlikely to explain the variation. In a previously published study, IMR was found to be independent of heart rate, contractility, and loading conditions [21]. Furthermore, we found no significant differences in coronary pressure levels between repeated measurements.

Given the limited duration of steady-state maximal hyperemia following IC adenosine injection, accurate repeated transit time recordings also require timing. There is an obvious risk of obtaining longer transit times as hyperemia attenuates, thereby driving the subsequently calculated IMR values towards a higher level. In the present study, we assessed hyperemia visually from the level of *P*_*d*_/*P*_*a*_ and it cannot be ruled out that the second and third transit time recordings after IC adenosine administration were obtained during near but not maximal hyperemia. However, we observed a comparable statistical spreading of the transit times following IC and IV adenosine administration. The dispersion of the transit times was low both for IC and IV adenosine.

Calculation of IMR has classically been derived from the mean of three hyperemic transit time values [4]. Repeated measurements carry the advantage of reducing the impact of extreme outliers. In order to simplify the procedure, we speculate if IMR calculated with a transit time value based on a single saline bolus injection after IC adenosine administration would be comparable to that calculated with *T*_*mn*_ based on 3 bolus injections during IV adenosine infusion. Our findings show an excellent agreement suggesting that a single bolus injection of saline during IC adenosine-induced hyperemia may be a potential alternative to the classic method based on 3 bolus injections during IV adenosine infusion. Our data suggest that an IMR value below the threshold of 25 with this simplified algorithm would be highly predictive of a “true normal” IMR as derived from hyperemic *T*_*mn*_ based on 3 saline injections. In case of a pathological result from a single saline bolus injection (i.e., an IMR > 25), additional transit time recordings may be performed in order to rule out false positive results. Such a hybrid approach has to be validated in a large cohort including a wide spectrum of IMR values but offers a simplified, time-saving, and potentially cost-saving way of evaluating coronary microvascular resistance. Previous studies have evaluated alternative hyperemic agents for intracoronary pressure-dilution and thermodilution measurements. As an example, intracoronary injection of Nicorandil (carrying the potential advantage of longer hyperemia duration as compared to IC adenosine) was proven safe and effective in inducing hyperemia for the calculation of FFR [22, 23] as well as IMR [23] when compared to IV adenosine. To our knowledge, comparison between IC nicorandil and IC adenosine for the calculation of IMR has not been studied.

### 4.1. Limitations

The main limitation of our analysis is the low number of subjects included. No previous data were available for sample size calculation, and the analysis has to be considered of exploratory nature. In 84% of the cases, measurements were performed in the left anterior descending artery where the steady-state plateau of IC adenosine-induced hyperemia has been shown to be longer than in the right coronary artery [15]. Therefore, it is uncertain if our results can be generalized to the measurement of *T*_*mn*_ in the right coronary artery. Nevertheless, we believe that our results represent an important hypothesis generating base for future research. Measurements were performed by two different operators, and no interobserver variability was assessed. Intraobserver variability, however, seems to be low when measurements are repeated within a short time interval. In our analysis, a vast majority of the subjects were found to have IMR values within the previously suggested normal range. It cannot be ruled out that the level of agreement between measurements during IC and IV adenosine administration is higher in the normal range of IMR and caution should therefore be taken extrapolating the results to a population of patients with predominantly pathological values (i.e., > 25). The measurement performance time and rate of side effects for each administration route were not systematically registered and are therefore not possible to make accurate comparisons on these aspects.

## 5. Conclusions

Calculation of IMR using IC adenosine seems feasible and comparable to standard measurements during IV adenosine infusion. Variation of transit times within a patient was found to be lower when using IC adenosine, translating into higher repeatability of IMR as compared to measurements with IV adenosine. Although our results remain to be validated in larger cohorts, the use of IC adenosine may facilitate routine invasive assessment of the coronary microcirculation, potentially saving time and cost as well as reducing patient discomfort.

## Figures and Tables

**Table 1:** Baseline characteristics.

Age, years	64.1	±7.7
Male gender	20	(64.5%)
Smoking habit	1	(3.2%)
Hypertension	20	(64.5%)
Diabetes mellitus	10	(32.3%)
Hyperlipidemia	19	(61.3%)
Atrial fibrillation	3	(9.7%)
BMI, kg/m^2^	25.9	±3.8
HR, bpm	64.5	(57.2–75.5)
BP systolic, mmHg	134	±18.81
BP diastolic, mmHg	75	(70.5–80)
Renal insufficiency (GFR < 60 ml/min/1,73 m^2^)	4	(12.5%)
Total cholesterol, mmol/L	3.9	±1.26
LDL-cholesterol, mmol/L	1.99	±1.02
HbA1c, mmol/mol	43	(37–50)
LVEF, %	56	(55–60)
Statins	25	(80.6%)
ACE inh or ARB-II	24	(77.4%)
Beta-blockers	8	(25.8%)
Oral antidiabetic agents	10	(32.3%)
Insulin	6	(19.4%)
Radial access	30	(96.8%)
Two vessel disease	9	(29.0%)
Three vessel disease	5	(16.1%)
LAD	26	(83.9%)
RCA	3	(9.7%)
LCX	2	(6.5%)
Syntax score	13.2	±7.06
Functionally significant stenosis (FFR ≤ 0, 80)	15	(48.4%)

BMI, body mass index; HR, heart rate; BP, blood pressure; GFR, glomerular filtration rate; LDL, low-density lipoprotein-cholesterol; HbA1c, glycated hemoglobin; LVEF, left ventricular ejection fraction; ACE-inh, angiotensin-converting enzyme inhibitors; ARB-II, angiotensin II receptor blockers; LAD, left anterior descending artery; RCA, right coronary artery; LCX, left circumflex artery; FFR, fractional flow reserve.

**Table 2:** Individual values of mean transit time and standard deviation for each subject obtained with intracoronary and intravenous adenosine, respectively.

IC		IV	
Mean	SD	Mean	SD
0.42	0.08	0.48	0.19
0.48	0.14	0.45	0.03
0.41	0.16	0.34	0.12
0.16	0.02	0.14	0.02
0.14	0.02	0.23	0.08
0.17	0.07	0.19	0.02
0.13	0.01	0.11	0.01
0.15	0.02	0.12	0.02
0.22	0.06	0.21	0.02
0.33	0.04	0.37	0.05
0.27	0.02	0.21	0.01
0.19	0.02	0.20	0.03
0.39	0.12	0.40	0.06
0.25	0.05	0.18	0.01
0.11	0.01	0.16	0.07
0.11	0.01	0.13	0.03
0.15	0.05	0.14	0.05
0.12	0.03	0.15	0.07
0.13	0.02	0.13	0.03
0.12	0.01	0.13	0.01
0.14	0.01	0.17	0.03
0.19	0.02	0.20	0.08
0.23	0.03	0.25	0.13
0.43	0.04	0.36	0.07
0.13	0.02	0.11	0.00
0.16	0.04	0.11	0.07
0.15	0.04	0.24	0.10
0.21	0.04	0.27	0.08
0.16	0.05	0.19	0.02
0.15	0.04	0.29	0.09
0.17	0.00	0.22	0.02
**0.16**	**0.14–0.24**	**0.20**	**0.14–0.27**

Values are given in seconds. IC, intracoronary adenosine; IV, intravenous adenosine; SD, standard deviation.

## Data Availability

The data, analytic methods, and study materials have been made available to other researchers for purposes of reproducing the results or replicating the procedure. The complete supporting data on coronary pressure and coronary flow are displayed in the Supporting Information (Tables S.1 and S.2). The data corresponding to repeated measures subanalysis are available from the corresponding author upon request.

## Supplementary Materials

Supplementary Materials
Figure S.1 shows a Bland–Altman diagram of the agreement between the transit time obtained with the first injection of saline during IC adenosine-induced hyperemia and *T*_*mn*_ obtained with IV adenosine. Figure S.2 represents a Bland–Altman diagram showing agreement between IMR calculated with the transit time obtained from the first injection of saline during IC adenosine-induced hyperemia and the *T*_*mn*_ obtained with IV adenosine. Table S.1 lists all discrete and average transit times at rest and during hyperemia for IC and IV adenosine. Finally, Table S.2 makes available all data on pressure and Thermodilution measurements with IC adenosine (Table S.2A) and IV adenosine (Table S.2B).
