# Supplementary material for: Index of Microcirculatory Resistance Measured during Intracoronary Adenosine-Induced Hyperemia
Source: J Interv Cardiol. 2020 May 12;2020:4829647. doi: 10.1155/2020/4829647 (PMC7243016; doi:10.1155/2020/4829647)
Supplement: Supplementary Materials — Figure S.1 shows a Bland–Altman diagram of the agreement between the transit time obtained with the first injection of saline during IC adenosine-induced hyperemia and Tmn obtained with IV adenosine. Figure S.2 represents a Bland–Altman diagram showing agreement between IMR calculated with the transit time obtained from the first injection of saline during IC adenosine-induced hyperemia and the Tmn obtained with IV adenosine. Table S.1 lists all discrete and average transit times at rest and during hyperemia for IC and IV adenosine. Finally, Table S.2 makes available all data on pressure and Thermodilution measurements with IC adenosine (Table S.2A) and IV adenosine (Table S.2B). [file 4829647.f1.docx]

**SUPPLEMENTARY MATERIAL**


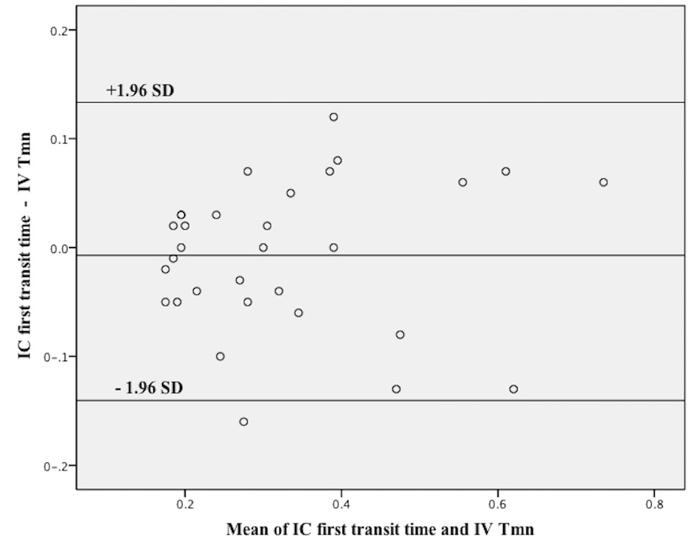


Figure S.1 Bland Altman diagram showing agreement between the transit time obtained with the first injection of saline during intracoronary adenosine-induced hyperemia and T_mn_ obtained with intravenous adenosine. Abbreviations: T_mn,_ mean transit time; IC, intracoronary adenosine; IV, intravenous adenosine; SD, standard deviation.


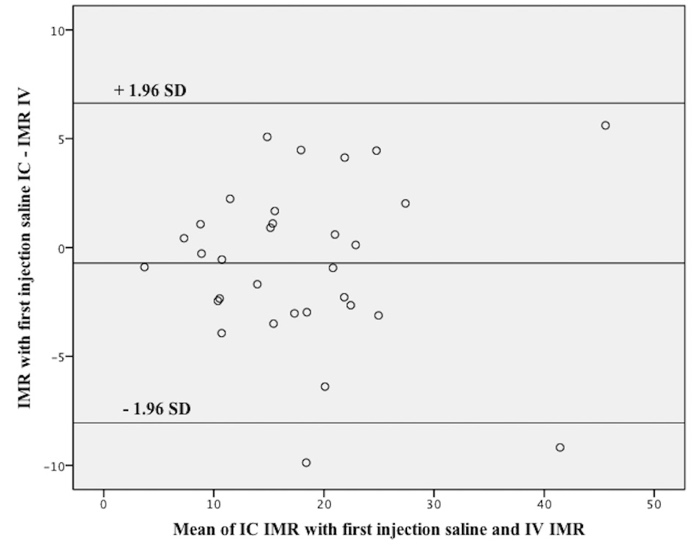


Figure S.2 Bland Altman diagram showing agreement between IMR calculated with the transit time obtained from the first injection of saline during intracoronary adenosine-induced hyperemia and the T_mn_ obtained with intravenous adenosine. Abbreviations: IMR, Index of Microcirculatory Resistance; T_mn,_ mean transit time; IC, intracoronary adenosine; IV, intravenous adenosine; SD, standard deviation.

|  | IC adenosine | | | | | | | | IV adenosine | | | | | | | |
| --- | --- | --- | --- | --- | --- | --- | --- | --- | --- | --- | --- | --- | --- | --- | --- | --- |
|  | REST | | | | HYPEREMIA | | | | REST | | | | HYPEREMIA | | | |
| SUBJ | TT1 | TT2 | TT3 | Tmn | TT1 | TT2 | TT3 | Tmn | TT1 | TT2 | TT3 | Tmn | TT1 | TT2 | TT3 | Tmn |
| 1 | 0.26 | 0.15 | 0.26 | 0.22 | 0.11 | 0.12 | 0.11 | 0.11 | 0.23 | 0.22 | 0.25 | 0.23 | 0.12 | 0.11 | 0.24 | 0.16 |
| 2 | 0.34 | 0.71 | 0.34 | 0.46 | 0.12 | 0.11 | 0.11 | 0.11 | 0.41 | 0.44 | 0.65 | 0.50 | 0.13 | 0.16 | 0.11 | 0.13 |
| 3 | 1.59 | 1.07 | 0.82 | 1.16 | 0.21 | 0.12 | 0.13 | 0.16 | 1.03 | 1.16 | 1.18 | 1.13 | 0.20 | 0.10 | 0.13 | 0.14 |
| 4 | 1.06 | 1.37 | 1.54 | 1.32 | 0.10 | 0.11 | 0.15 | 0.12 | 1.49 | 1.42 | 1.07 | 1.33 | 0.11 | 0.23 | 0.12 | 0.15 |
| 5 | 0.65 | 0.51 | 0.56 | 0.57 | 0.11 | 0.14 | 0.14 | 0.13 | 0.51 | 0.35 | 0.32 | 0.39 | 0.16 | 0.12 | 0.10 | 0.13 |
| 6 | 0.25 | 0.21 | 0.18 | 0.22 | 0.13 | 0.13 | 0.11 | 0.12 | 0.13 | 0.10 | 0.14 | 0.12 | 0.13 | 0.13 | 0.14 | 0.13 |
| 7 | 0.92 | 0.32 | 0.48 | 0.57 | 0.13 | 0.13 | 0.15 | 0.14 | 0.80 | 0.55 | 0.65 | 0.67 | 0.20 | 0.15 | 0.15 | 0.17 |
| 8 | 0.62 | 0.57 | 0.55 | 0.58 | 0.20 | 0.19 | 0.17 | 0.19 | 0.48 | 0.32 | 0.58 | 0.46 | 0.11 | 0.23 | 0.25 | 0.20 |
| 9 | 1.09 | 1.14 | 1.00 | 1.08 | 0.26 | 0.21 | 0.21 | 0.22 | 1.21 | 1.00 | 1.15 | 1.12 | 0.16 | 0.20 | 0.40 | 0.26 |
| 10 | 1.96 | 1.28 | 1.90 | 1.71 | 0.43 | 0.40 | 0.47 | 0.43 | 2.00 | 1.86 | 1.57 | 1.81 | 0.44 | 0.31 | 0.32 | 0.36 |
| 11 | 0.72 | 0.53 | 0.34 | 0.53 | 0.13 | 0.12 | 0.15 | 0.13 | 0.50 | 0.24 | 0.13 | 0.29 | 0.11 | 0.11 | 0.11 | 0.11 |
| 12 | 0.46 | 0.80 | 0.39 | 0.55 | 0.14 | 0.21 | 0.14 | 0.16 | 0.78 | 0.47 | 0.78 | 0.68 | 0.07 | 0.19 | 0.07 | 0.11 |
| 13 | 0.49 | 0.27 | 0.41 | 0.39 | 0.20 | 0.13 | 0.13 | 0.15 | 0.64 | 0.77 | 0.32 | 0.57 | 0.33 | 0.14 | 0.25 | 0.24 |
| 14 | 1.00 | 0.64 | 0.93 | 0.85 | 0.21 | 0.24 | 0.17 | 0.21 | 0.85 | 0.79 | 1.15 | 0.93 | 0.24 | 0.20 | 0.36 | 0.27 |
| 15 | 1.95 | 1.10 | 1.39 | 1.48 | 0.21 | 0.16 | 0.11 | 0.16 | 1.26 | 1.09 | 1.12 | 1.16 | 0.19 | 0.18 | 0.21 | 0.19 |
| 16 | 1.62 | 1.46 | 1.45 | 1.51 | 0.13 | 0.19 | 0.12 | 0.15 | 1.21 | 1.47 | 1.54 | 1.41 | 0.19 | 0.31 | 0.36 | 0.29 |
| 17 | 0.34 | 0.23 | 0.28 | 0.28 | 0.17 | 0.17 | 0.17 | 0.17 | 0.79 | 0.77 | 0.92 | 0.83 | 0.24 | 0.21 | 0.21 | 0.22 |
| 18 | 1.41 | 0.73 | 1.01 | 1.05 | 0.37 | 0.37 | 0.51 | 0.42 | 0.53 | 0.65 | 0.52 | 0.53 | 0.27 | 0.54 | 0.64 | 0.50 |
| 19 | 0.38 | 0.70 | 0.96 | 0.68 | 0.51 | 0.60 | 0.32 | 0.48 | 0.74 | 0.86 | 0.68 | 0.76 | 0.49 | 0.43 | 0.43 | 0.45 |
| 20 | 1.17 | 0.48 | 1.12 | 0.92 | 0.39 | 0.26 | 0.57 | 0.41 | 0.39 | 0.80 | 0.30 | 0.49 | 0.20 | 0.42 | 0.39 | 0.33 |
| 21 | 0.27 | 0.15 | 0.20 | 0.21 | 0.17 | 0.14 | 0.17 | 0.16 | 0.37 | 0.41 | 0.53 | 0.43 | 0.15 | 0.15 | 0.12 | 0.14 |
| 22 | 0.25 | 0.22 | 0.41 | 0.29 | 0.13 | 0.14 | 0.16 | 0.15 | 0.29 | 0.11 | 0.54 | 0.31 | 0.19 | 0.18 | 0.32 | 0.23 |
| 23 | 0.40 | 0.28 | 0.41 | 0.40 | 0.24 | 0.10 | 0.18 | 0.17 | 0.41 | 0.30 | 0.30 | 0.34 | 0.21 | 0.19 | 0.18 | 0.19 |
| 24 | 0.60 | 0.27 | 0.28 | 0.39 | 0.14 | 0.12 | 0.14 | 0.13 | 0.27 | 0.45 | 0.41 | 0.38 | 0.12 | 0.11 | 0.10 | 0.11 |
| 25 | 0.83 | 0.60 | 0.50 | 0.64 | 0.14 | 0.17 | 0.13 | 0.15 | 0.50 | 0.43 | 0.46 | 0.46 | 0.11 | 0.14 | 0.10 | 0.12 |
| 26 | 0.89 | 0.66 | 0.84 | 0.80 | 0.28 | 0.17 | 0.20 | 0.22 | 0.36 | 0.39 | 0.37 | 0.37 | 0.20 | 0.23 | 0.20 | 0.21 |
| 27 | 1.51 | 2.67 | 1.97 | 2.05 | 0.29 | 0.36 | 0.35 | 0.33 | 0.60 | 1.84 | 1.02 | 1.15 | 0.32 | 0.37 | 0.41 | 0.37 |
| 28 | 1.21 | 1.08 | 0.86 | 1.05 | 0.29 | 0.28 | 0.25 | 0.27 | 0.19 | 0.20 | 0.38 | 0.26 | 0.22 | 0.21 | 0.20 | 0.21 |
| 29 | 0.56 | 0.29 | 0.32 | 0.39 | 0.17 | 0.19 | 0.21 | 0.19 | 0.36 | 0.20 | 0.55 | 0.37 | 0.20 | 0.23 | 0.18 | 0.20 |
| 30 | 0.80 | 1.00 | 0.93 | 0.91 | 0.27 | 0.50 | 0.41 | 0.39 | 0.64 | 0.30 | 1.06 | 0.67 | 0.45 | 0.34 | 0.42 | 0.40 |
| 31 | 0.73 | 0.61 | 0.57 | 0.64 | 0.30 | 0.22 | 0.22 | 0.24 | 0.81 | 0.44 | 0.43 | 0.56 | 0.17 | 0.18 | 0.19 | 0.18 |
| **M** | **0.73** | **0.61** | **0.56** | **0.64** | **0.20** | **0.17** | **0.17** | **0.16** | **0.53** | **0.45** | **0.55** | **0.53** | **0.19** | **0.19** | **0.20** | **0.20** |
| **IQR** | **0.4-1.17** | **0.28-1.07** | **0.34-1.0** | **0.39-1.05** | **0.13-0.28** | **0.13-0.24** | **0.13-0.22** | **0.16-0.24** | **0.37-0.81** | **0.30-0.86** | **0.37-1.06** | **0.37-0.93** | **0.13-0.24** | **0.14-0.23** | **0.12-0.36** | **0.14-0.27** |

Table S.1 – Transit times

Discrete and average transit times at rest and during hyperemia for IC and IV adenosine. Abbreviations: IC, intracoronary adenosine; IV, intravenous adenosine; SUBJ, subject; TT1, first transit time; TT2, second transit time; TT3, third transit time; Tmn, mean transit time; M, median; IQR, interquartile range (25^th^-75^th^).

1. **IC ADENOSINE**

|  | REST | | | | HYPEREMIA | | | |  |  |
| --- | --- | --- | --- | --- | --- | --- | --- | --- | --- | --- |
| SUBJ | Pa | Pd | Pd/Pa | Tmn | Pa | Pd | FFR | Tmn | CFR | IMR |
| 1 | 64 | 58 | 0.91 | 0.22 | 66 | 53 | 0.80 | 0.11 | 2.0 | 5.8 |
| 2 | 80 | 72 | 0.91 | 0.46 | 64 | 52 | 0.81 | 0.11 | 4.2 | 5.7 |
| 3 | 85 | 78 | 0.91 | 1.16 | 79 | 64 | 0.81 | 0.16 | 7.3 | 10.2 |
| 4 | 94 | 88 | 0.94 | 1.32 | 72 | 61 | 0.84 | 0.12 | 11.0 | 7.3 |
| 5 | 81 | 74 | 0.91 | 0.57 | 62 | 53 | 0.85 | 0.13 | 4.4 | 6.9 |
| 6 | 98 | 92 | 0.94 | 0.22 | 93 | 80 | 0.86 | 0.12 | 1.8 | 9.6 |
| 7 | 90 | 85 | 0.95 | 0.57 | 102 | 87 | 0.86 | 0.14 | 4.1 | 12.2 |
| 8 | 89 | 82 | 0.93 | 0.58 | 81 | 71 | 0.87 | 0.19 | 3.1 | 13.5 |
| 9 | 71 | 71 | 1.00 | 1.08 | 68 | 60 | 0.87 | 0.22 | 4.9 | 13.2 |
| 10 | 93 | 86 | 0.93 | 1.71 | 86 | 75 | 0.87 | 0.43 | 4.0 | 32.3 |
| 11 | 93 | 86 | 0.93 | 0.53 | 96 | 84 | 0.88 | 0.13 | 4.1 | 10.9 |
| 12 | 71 | 64 | 0.91 | 0.55 | 68 | 60 | 0.88 | 0.16 | 3.4 | 9.6 |
| 13 | 82 | 76 | 0.93 | 0.39 | 78 | 69 | 0.89 | 0.15 | 2.6 | 10.4 |
| 14 | 120 | 117 | 0.97 | 0.85 | 125 | 117 | 0.93 | 0.21 | 4.0 | 24.6 |
| 15 | 79 | 78 | 0.99 | 1.48 | 72 | 67 | 0.94 | 0.16 | 9.3 | 10.7 |
| 16 | 100 | 100 | 1.00 | 1.51 | 72 | 69 | 0.96 | 0.15 | 10.1 | 10.4 |
| 17 | 86 | 87 | 1.01 | 0.28 | 90 | 90 | 1.00 | 0.17 | 1.6 | 15.3 |
| 18 | 92 | 75 | 0.82 | 1.05 | 81 | 45 | 0.55 | 0.42 | 2.5 | 9.7 |
| 19 | 73 | 61 | 0.83 | 0.68 | 56 | 32 | 0.57 | 0.48 | 1.4 | 8.4 |
| 20 | 83 | 69 | 0.84 | 0.92 | 71 | 43 | 0.60 | 0.41 | 2.2 | 14.3 |
| 21 | 75 | 63 | 0.84 | 0.21 | 75 | 51 | 0.68 | 0.16 | 1.3 | 5.2 |
| 22 | 79 | 68 | 0.86 | 0.29 | 67 | 46 | 0.69 | 0.15 | 1.9 | 2.9 |
| 23 | 93 | 85 | 0.92 | 0.40 | 120 | 84 | 0.70 | 0.17 | 2.4 | 5.4 |
| 24 | 78 | 70 | 0.90 | 0.39 | 78 | 55 | 0.71 | 0.13 | 3.0 | 6.5 |
| 25 | 79 | 71 | 0.91 | 0.64 | 72 | 52 | 0.71 | 0.15 | 4.3 | 6.9 |
| 26 | 107 | 98 | 0.91 | 0.80 | 106 | 76 | 0.71 | 0.22 | 3.6 | 14.9 |
| 27 | 89 | 83 | 0.94 | 2.05 | 73 | 54 | 0.74 | 0.33 | 6.2 | 9.9 |
| 28 | 71 | 61 | 0.87 | 1.05 | 78 | 60 | 0.76 | 0.27 | 3.9 | 14.9 |
| 29 | 103 | 94 | 0.92 | 0.39 | 98 | 75 | 0.76 | 0.19 | 2.1 | 10.6 |
| 30 | 67 | 63 | 0.94 | 0.91 | 58 | 45 | 0.77 | 0.39 | 2.3 | 16.3 |
| 31 | 105 | 100 | 0.96 | 0.64 | 80 | 63 | 0.78 | 0.24 | 2.7 | 9.3 |
|  | **86** | **79** | **0.92** | **0.77** | **78** | **64** | **0.80** | **0.16** | **3.4** | **10.2** |
|  | **13** | **14** | **0.05** | **0.47** | **68-90** | **17** | **0.11** | **0,14-0,24** | **2.2-4.2** | **6.9-13.5** |

1. **IV ADENOSINE**

|  | REST | | | | HYPEREMIA | | | |  |  |
| --- | --- | --- | --- | --- | --- | --- | --- | --- | --- | --- |
| SUBJ | Pa | Pd | Pd/Pa | Tmn | Pa | Pd | FFR | Tmn | CFR | IMR |
| 1 | 75 | 67 | 0.90 | 0.23 | 71 | 61 | 0.81 | 0.16 | 1.4 | 9.8 |
| 2 | 74 | 65 | 0.89 | 0.50 | 75 | 66 | 0.88 | 0.13 | 3.8 | 8.6 |
| 3 | 86 | 80 | 0.93 | 1.13 | 78 | 64 | 0.82 | 0.14 | 8.1 | 9.0 |
| 4 | 94 | 89 | 0.95 | 1.33 | 67 | 57 | 0.84 | 0.15 | 8.9 | 8.6 |
| 5 | 86 | 79 | 0.90 | 0.39 | 56 | 47 | 0.85 | 0.13 | 3.0 | 6.1 |
| 6 | 104 | 98 | 0.94 | 0.12 | 79 | 73 | 0.92 | 0.13 | 0.9 | 9.5 |
| 7 | 108 | 101 | 0.94 | 0.67 | 95 | 84 | 0.88 | 0.17 | 3.9 | 14.3 |
| 8 | 92 | 85 | 0.93 | 0.46 | 78 | 68 | 0.87 | 0.20 | 2.3 | 13.6 |
| 9 | 69 | 68 | 0.99 | 1.12 | 82 | 72 | 0.88 | 0.26 | 4.3 | 18.7 |
| 10 | 104 | 97 | 0.94 | 1.81 | 88 | 74 | 0.84 | 0.36 | 5.0 | 26.6 |
| 11 | 110 | 100 | 0.91 | 0.29 | 98 | 84 | 0.86 | 0.11 | 2.6 | 9.2 |
| 12 | 76 | 70 | 0.93 | 0.68 | 65 | 56 | 0.86 | 0.11 | 6.2 | 6.2 |
| 13 | 99 | 93 | 0.94 | 0.57 | 75 | 67 | 0.89 | 0.24 | 2.4 | 16.1 |
| 14 | 128 | 125 | 0.97 | 0.93 | 133 | 125 | 0.94 | 0.27 | 3.4 | 33.8 |
| 15 | 82 | 82 | 1.00 | 1.16 | 93 | 88 | 0.94 | 0.19 | 6.1 | 16.7 |
| 16 | 94 | 94 | 1.00 | 1.41 | 68 | 65 | 0.96 | 0.29 | 4.9 | 18.9 |
| 17 | 88 | 90 | 1.02 | 0.83 | 69 | 69 | 0.99 | 0.22 | 3.8 | 15.2 |
| 18 | 100 | 77 | 0.77 | 0.53 | 85 | 48 | 0.56 | 0.50 | 1.1 | 24.0 |
| 19 | 72 | 59 | 0.83 | 0.76 | 66 | 38 | 0.57 | 0.45 | 1.7 | 17.1 |
| 20 | 85 | 70 | 0.83 | 0.49 | 82 | 51 | 0.62 | 0.33 | 1.5 | 16.8 |
| 21 | 81 | 70 | 0.86 | 0.43 | 84 | 57 | 0.69 | 0.14 | 3.1 | 8.0 |
| 22 | 88 | 75 | 0.86 | 0.31 | 58 | 40 | 0.69 | 0.23 | 1.3 | 9.2 |
| 23 | 101 | 93 | 0.91 | 0.34 | 77 | 58 | 0.76 | 0.19 | 1.8 | 11.0 |
| 24 | 80 | 73 | 0.91 | 0.38 | 61 | 53 | 0.87 | 0.11 | 3.5 | 5.8 |
| 25 | 84 | 75 | 0.90 | 0.46 | 68 | 52 | 0.76 | 0.12 | 3.8 | 6.2 |
| 26 | 112 | 102 | 0.92 | 0.37 | 104 | 80 | 0.77 | 0.21 | 1.8 | 16.8 |
| 27 | 81 | 74 | 0.92 | 1.15 | 76 | 54 | 0.71 | 0.37 | 3.1 | 20.0 |
| 28 | 83 | 63 | 0.76 | 0.26 | 76 | 56 | 0.74 | 0.21 | 1.2 | 11.8 |
| 29 | 111 | 100 | 0.90 | 0.37 | 100 | 83 | 0.83 | 0.20 | 1.9 | 16.6 |
| 30 | 65 | 60 | 0.92 | 0.67 | 57 | 42 | 0.74 | 0.40 | 1.7 | 16.8 |
| 31 | 92 | 86 | 0.93 | 0.56 | 83 | 63 | 0.76 | 0.18 | 3.1 | 11.3 |
|  | **90** | **83** | **0.91** | **0.53** | **79** | **64** | **0.81** | **0.20** | **3.1** | **11.8** |
|  | **15** | **15** | **0.06** | **0,37-0,93** | **16** | **17** | **0.11** | **0,14-0,27** | **1.7-4.0** | **8.5-16** |

Table S.2: Pressure and Thermodilution measurements with IC adenosine (A) and IV adenosine (B). Abbreviations: IC, intracoronary adenosine; IV, intravenous adenosine; SUBJ, subject; Pa, aortic pressure; Pd, distal coronary pressure; Tmn, mean transit time; FFR, fractional flow reserve; CFR, coronary flow reserve; IMR, Index of Microcirculatory Resistance.
